# Effectiveness of Oral Sensory-Motor Stimulation in Premature Infants in the Neonatal Intensive Care Unit (NICU) Systematic Review

**DOI:** 10.3390/children8090758

**Published:** 2021-08-31

**Authors:** Paula Rodriguez Gonzalez, Veronica Perez-Cabezas, Gema Chamorro-Moriana, Carmen Ruiz Molinero, Ana María Vazquez-Casares, Gloria Gonzalez-Medina

**Affiliations:** 1 Comprehensive Care Center, COCEMFE, Campos Góticos, s/n, 24005 Leon, Spain; paula.rodrigezgon@alum.uca.es; 2 Department of Nursing and Physiotherapy, Faculty of Nursing and Physiotherapy, University of Cadiz, Avda. Ana de Viya, 52, 11009 Cadiz, Spain; carmen.ruizmolinero@uca.es (C.R.M.); gloriagonzalez.medina@uca.es (G.G.-M.); 3 Investigation Group, [CTS1038] eMpOwering Health by Physical Activity, Exercise and Nutrition, University of Cadiz, 11009 Cadiz, Spain; 4 Department of Physiotherapy, University of Seville, Avenzoar, 6, 41009 Seville, Spain; gchamorro@us.es; 5 Department of Nursing and Physiotherapy, University of Leon-Campus of Vegazana, 24007 Leon, Spain; amvc@unileon.es; 6 Investigation Group CTS-986, Physical Therapy and Health (FISA), University Institute of Research in Social Sustainable Development (INDESS), University of Cadiz, 11009 Cadiz, Spain

**Keywords:** premature birth, infant, premature, mouth, physical therapy modalities, physical therapist assistants

## Abstract

The aim of this study was to identify and to assess the best evidence currently available on the effectiveness of oral sensory-motor stimulation in preterm infants in the neonatal intensive care unit. We performed a systematic review following the Preferred Reporting Items for Systematic Reviews (PRISMA) statements. The search was conducted using the Pubmed, Web of Science (WOS), PEDro and Scopus databases. Clinical trials were reviewed and PEDro rating scale was used to assess the methodological quality of these studies. Results: 1267 studies were found and 11 were relevant and included in this review. Improvements were obtained in achieving independent feeding, maturation of the sucking pattern, transition to full feeding, motor function and length of hospital stay in most studies. Conclusions: there is evidence to support the benefits of the use of oral sensorimotor stimulation to achieve independent oral feeding in preterm infants, thereby reducing their stay in the Neonatal Intensive Care Unit.

## 1. Introduction

According to the World Health Organization, approximately 15 million babies are born prematurely every year, 8% to 10% of them in industrialized countries [1].

Pre-birth factors [2,3] combined with premature birth risk factors [4,5,6] increase the risk of death, which is estimated at one million babies dying in the first year of life [4,5,6,7,8]. Those who survive, approximately 50%, have developmental functional diversity such as motor, cognitive and behavioral impairments. More specifically, they may experience feeding difficulties, infections, jaundice, apnea of prematurity and retinopathy of prematurity [1]. Moreover, some of them are not limited only to the perinatal period, but they can be extended throughout life generating great disability and impact on the well-being of the babies [7,8,9,10].

In order to avoid these complications as much as possible and to ensure the extra-uterine development during the last weeks of pregnancy, we have the Neonatal Intensive Care Unit of the Hospital [NICU]. Premature babies remain in this unit until the time of discharge from hospital, thus completing their development. These units increase survival, however, babies are exposed to stressors and uncomfortable and painful procedures, which lead to structural and functional changes in specific areas of the brain [9]. All this will have negative effects on proper growth and development, both neurological and psychological [11,12].

Currently, “Newborn Individualized Developmental Care and Assessment Program” is being sought, the main objective of which is to achieve the adequate neurological and emotional development of the child by adapting the environment [13]. To this end, care actions based on somatic stimulation -stimulation of the somatosensory system-, kinesthetic -movement stimulation- and sensory -stimulation of the senses: visual, auditory, tactile, olfactory and taste stimulation- have been introduced [14,15].

Knowledge and education as a nurse about infant feeding is very important [16]. Knowing all the elements that take part in its improvement is fundamental to carry out coordinated care with the rest of the interdisciplinary team [17].

Until relatively recently physical therapy, within the NICU, focused mainly on the handling and postural care of babies, as well as exercises at the respiratory system to eliminate secretions. However, thanks to the implementation of “developmental care”, the role of the physical therapist has incorporated the promotion of sensorimotor development [18], hence the importance of early intervention by the physical therapist to minimize the consequences of risk factors [19].

One of the roles of the physical therapist is to improve the baby’s oral motor control. The preterm infant has poor oral motor control related, in part, to weaker muscle tone around the mouth, less sensitivity and less tongue strength compared to the full-term infant [20]. A deficit at this level can increase the length of stay in hospital, as the energy expenditure involved in not being able to feed properly results in delayed motor development [21]. For this reason, achieving efficient oral motor function should be a priority [22].

Oral sensory-motor stimulation is described as stroking or pressure on the peri- and intra-oral structures: the cheeks, lips, jaw, tongue, palate and gums, as well as non-nutritive suction of a pacifier [23,24]. In recent years, specific oral motor programs have been developed to increase functional strength and movement control [23]. Therefore, the Beckman Oral Motor Intervention [BOMI] [25] and the Premature Infant Oral Motor Intervention [PIOMI] [26] were developed to treat infants over and under 30 weeks of gestation, respectively. The protocol developed by Beckman has a total duration of 15 min. It uses mechanical, non-cognitively mediated muscle responses to seek responses to different stimuli. Different pressures, movements, ranges of motion, force and control of lip, cheek, jaw and tongue movement are applied. PIOMI is a 5-min oral motor intervention that provides assisted movement against resistance. It aims to activate muscle contraction and enhance strength. The goal of the intervention is to increase functional response to pressure and movement and control of lip, cheek, jaw and tongue movements. There are other methods of intervention, but they are already included in the two previous ones [27].

These methods show that activation of the oral area not only has results on the physiological function of the mouth and pharynx, but also facilitates growth and general neurological maturation [28,29]. Evidence reflects that an intervention at this level achieve an increase in oral intake, reduces the days of transition to full oral feeding and decreases the length of hospital stay. Stimulation of oral structures can trigger activation of the muscles responsible for controlling the head, neck and trunk, thus improving overall motor function. Furthermore, some studies have also shown that tactile sensorimotor input increases motor activity as a function of reflex responses and muscle tone as well as neurobehavioral organization in infants [11].

Nowadays, there are previous reviews that appear as a result of searches related to the subject of this article [27,30]. The objective of all of these reviews is assessing the effectiveness of oral sensorimotor stimulation in achieving complete oral feeding in a shorter time and a reducing length of stay in the hospital of preterm babies. In contrast to these articles, the present review aims to establish all the variables on which oral sensory-motor stimulation has an impact in premature infants admitted to the NICU.

The aim of this review is to identify and to evaluate, critically and objectively, the evidence currently available on the effectiveness of oral sensory-motor stimulation in preterm infants in the NICU.

## 2. Materials and Methods

### 2.1. Study Design

A systematic review was conducted following the Preferred Reporting Items for Systematic Reviews [PRISMA] [31] and Meta-analysis between January and March 2020. The databases PubMed, Scopus, Web of Science [WoS] and Physiotherapy Evidence Database [PEDro] were consulted.

This revision has been registered in PROSPERO with the code: 226833.

### 2.2. Search Strategy

The MeSH terms used were: “Premature Birth”, “Infant, Extremely Premature”, “Infant, Premature”, “Mouth”, “Physical Therapy Modalities”, “Physical Therapy Specialty”, “Physical Therapy Department, Hospital”, “Physical Therapist Assistants”. These descriptors were combined with the Boolean operators “OR” y “AND” (Table 1).

### 2.3. Criteria for Considering Studies

The search was limited to the title, abstract and keywords using the research question [PICO]:[32] (P) Patients with problems of interest: preterm patients born at less than 37 weeks gestation. (I) Intervention: Oral sensory and motor stimulation. (C) Comparison intervention: Compared against placebo or standard care programme. (O) Outcomes: length of hospital stay, transition time to full oral feeding, sucking skills, independent feeding skills, motor function and growth.

Inclusion criteria were established as follows: (a) Population: Patients born before 37 weeks of gestation. Male and female sex. Intervention performed in the Neonatal Intensive Care Unit. (b) Design: Clinical Trials [CTs]. (c) Language: English, Spanish. (d) Intervention: oral/mucosal sensorimotor stimulation.

Exclusion criteria were: (a) Population: Study subjects are in the family home with medical discharge. (b) Intervention: The intervention method of the study population included non-nutritive sucking but not oral/mucosal sensorimotor stimulation.

From each study, the variables related to the publication, as authors and year of publication, were obtained and reviewed. Variables related to the research carried out: sample size; age, sex and inclusion and exclusion criteria of the participants; main effects studied. In addition, we investigated the variables that were used to measure the effects and the applied intervention programs.

The variables evaluated in the different studies were those related to feed intake, volumes of feed consumed or lost, type of feeding, type of suction and growth control variables.

### 2.4. Assessment of the Methodological Quality of Studies

The methodological quality of the articles selected for this review was assessed using the PEDro scale [33]. This is a scale for the methodological assessment of randomised clinical trials involving physiotherapeutic intervention.

To guarantee the results, the assessment was performed independently by two investigators. Finally, the results were crosschecked. In case of discrepancy, a third investigator intervened.

### 2.5. Data Analysis

The meta-analysis of the data was performed using Review Manager 5.3 (The Nordic Cochrane Centre, The Cochrane Collaboration, (2014) [34] and EPIDAT software version 3.1 [35]. The first was used to check the homogeneity of the studies. Values were applied: I2 > 50%, indicating substantial heterogeneity, where randomized effect models were applied, and I2 < 50%, indicating substantial homogeneity, where the fixed effect model was applied. It was also used to obtain the results of the meta-analysis and the tables and graphs of forest and funnel plots. The second tool was used to check the risk of publication of the studies selected for meta-analysis. Whenever possible, Begg and Egger values were observed (*p* < 0.05).

## 3. Results

**Figure 1:**
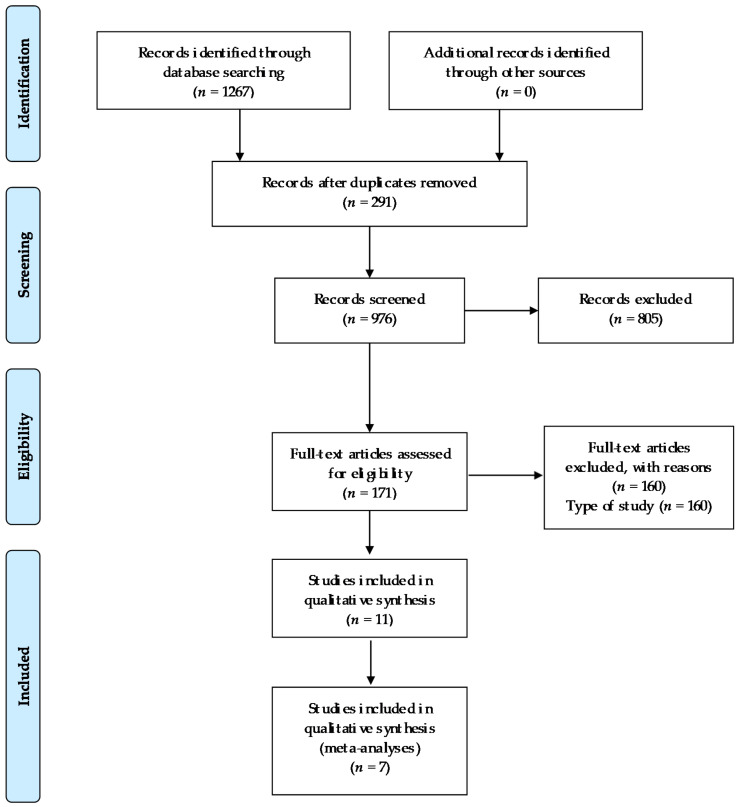
Flow chart.

Out of 1267 studies, 11 studies were selected for analysis in this review. The number of studies screened, assessed for eligibility, and included in the review, with reasons for exclusions, are reflected in the flow chart (Figure 1).

### 3.1. Data Extraction

The sample size of the different studies ranged from 19 to 75 participants. In total, the cumulative sample under study was 507 subjects. The sex of the participants, not all studies specify it, but in those that do, we found that the number of boys under study was 242, compared to 162 girls. The age range was between 26 and 34 weeks of gestation. The age of the babies at the start of the intervention protocol corresponds to the gestational age at birth.

Regarding the inclusion criteria, all infants were within the prenatal age range (less than 37 weeks’ gestation) and admitted to the Neonatal Intensive Care Unit. With the exception of two studies, [36,37] the rest specified that feeding at the time of initiation should be complete tube feeding.

Some studies [38,39,40] considered the absence of respiratory support at least 48 h before starting the intervention together with normal blood oxygen levels as inclusion criteria while those led by Fucile et al. [38,41,42,43] considered it necessary that the size of the subjects be appropriate to their gestational age. Hwang et al. [37] did not consider any of these criteria, with the exception of prenatal age which is common to all, but decided to include those infants with insufficient feeding (<4 mL of milk in the first 5 min). On the other hand, Ghomi et al. [36] established physiological stability at the time of oral stimulation, Apgar score of 6 and parental consent to participate as inclusion criteria for their study. Infants with congenital abnormalities or chronic disease were excluded from all studies. Information about the characteristics of the sample is given in more detail in Table 2.

To achieve independent feeding, different aspects were assessed: Overall intake (volume ingested/volume administered) and volume of milk taken during the first 5 min, volume of milk consumed in relation to session duration (mL/min) and volume of milk lost during feeding to assess feeding competence; [42] this ability was assessed by measuring the total volume of milk per spoon [39]. Other studies decided to establish two measures: the number of days it took for infants to achieve 30% independent oral feeding for the first 5 min, and the number of days it took to achieve 100% independent feeding, which they quantified as the oral milk intake (150 mL/kg/day) for three consecutive days [44]. Finally, the percentage of the prescribed volume ingested, mean volume ingested per suck (mL/suck) and time to complete feeding in minutes were measured [37].

Different parameters were chosen to assess the state of sucking ability in preterm infants: sucking frequency [37,42]; the measurement of non-nutritive sucking pressure through a dummy connected to a catheter [40] and the coordination between sucking-sucking and breathing, as well as the level of maturity of sucking pattern and skill development, measured with the Lau scale [42,45].

The transition time from tube feeding to independent feeding was also extensively studied [36,42,46]. Some researchers specify that they used a wati spoon [38] and a spoon [39] as a measuring tool.

The measurement of infant growth in relation to oral stimulation through weight gain was considered by three studies. Measuring the value at the end of the study [36,38] or daily [43].

The impact of the intervention programme on the motor function of preterm infants was measured using the Test of Infant Motor Performance [TIMP] [38,43] and the Neonatal Oro Motor Assessment Scale [NOMAS] [43].

The effects on the number of days of hospital stay of infants in the NICU before discharge were also widely considered [36,38,43,44,46].

Other variables were also studied to a lesser extent: breastfeeding skills, ref. [46] infant alertness, [37] physiological data (measurement of peripheral oxygen saturation levels [SpO2] and pulse rate [PR]) [37].

As for the oral sensorimotor intervention programs, these were variable in terms of the number of daily sessions, the time of application per session, the organization and method of application, and the overall application times (Table 2 and Table 3).

### 3.2. Assessment of the Methodological Quality of Studies

The total score on the PEDro scale ranged from 4 to 8 points. According to the results obtained, it can be seen that the greatest methodological deficit corresponds to the items referring to blinding of the therapists, hidden allocation of subjects and analysis by “intention to treat”, as in, almost none of the studies evaluated was a positive response obtained. Most of the clinical trials analysed scored 5/10 or less (Table 4).

### 3.3. Data Analysis and Outcomes

Of all the variables extracted from the studies included in this research, only two were included in the meta-analysis.

**Figure 2:**
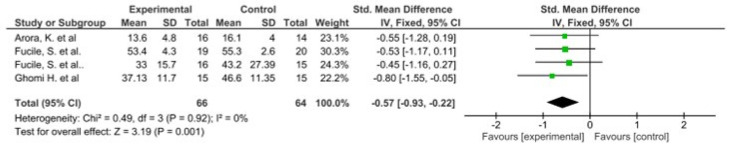
Forest plot panel of the comparison of the difference between the groups in duration of hospital stay.

**Figure 3:**
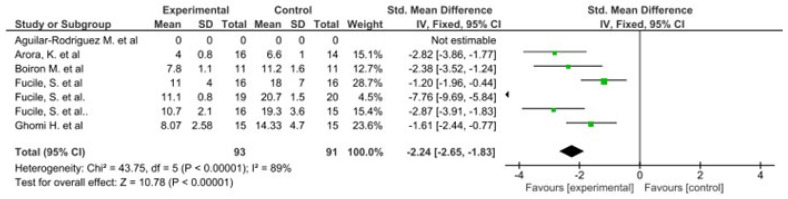
Forest plot panel of the comparison of the difference between the groups in number of days to reach full oral feeding.

The results of this meta-analysis indicate that there is only heterogeneity in the duration of hospital stay. In addition, statistically significant results are observed in favour of the intervention group in both variables (Figure 2 and Figure 3).

**Chart 1:**
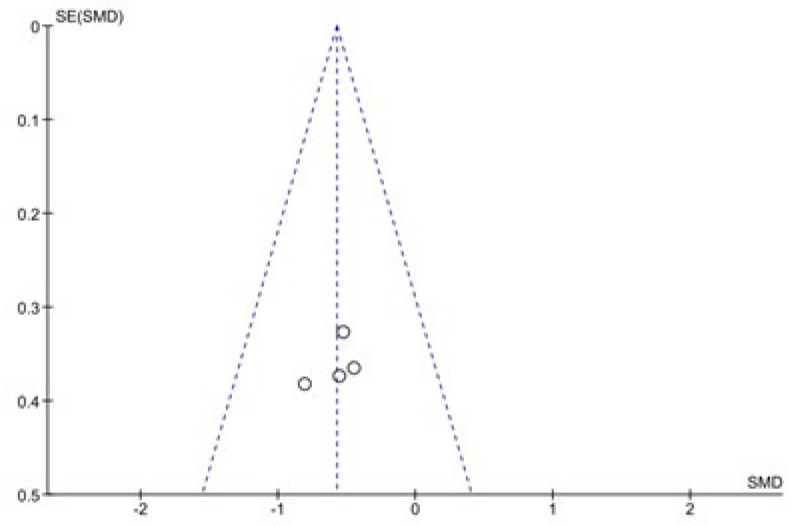
Funnel Plot of Duration of hospital stay.

**Chart 2:**
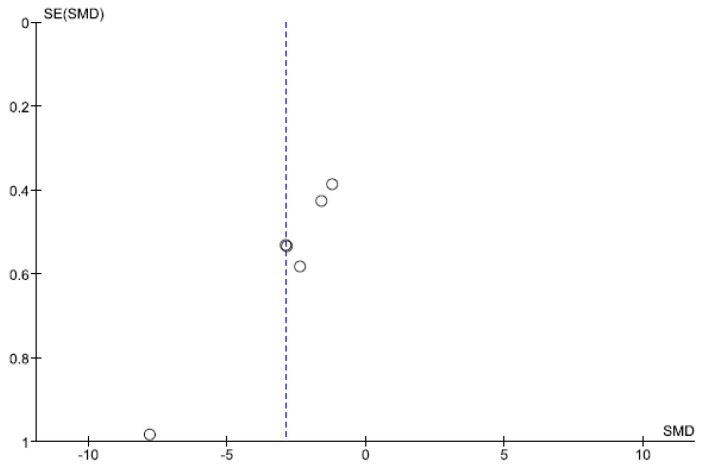
Funnel Plot of number of days to reach full oral feeding.

The results of the Begg and Egger tests indicate that there is only a risk of publication in the variable number of days to reach full oral feeding (Table 5). These results are confirmed by the funnel plots (Chart 1 and Chart 2).

## 4. Discussion

The systematic review of studies and meta-analysis of variables indicate that oral sensorimotor stimulation in the management of preterm infants hospitalized in the Neonatal Intensive Care Unit [NICU] is beneficial.

All of the reviewed studies have obtained results that reflect the benefits of oral sensorimotor stimulation in the management of premature infants. Although it is true that not all of them have studied its effects on the same variables, it is also true that not all of them have studied its effects on the same variables.

On the one hand, the main ones have been length of hospital stay, refs. [36,38,43,44,46] transition time from tube feeding to independent feeding, refs. [36,38,39,43,44,46] sucking skills, refs. [37,40,42,45] oral feeding skills, refs. [37,39,42,44] motor function [38,43] and growth [37,38,43]. On the other hand, some of them considered assessing the influence on breastfeeding skills, ref. [46] infants’ alertness [37] and their physiological constants [37]. This may be because the studies included in this research came from different countries. This makes the results quite generalisable. However, they reflect the heterogeneity of implementation that could be carried out in NICU.

The meta-analysis shows that the intervention oral sensory-motor stimulation is effective in premature infants in the NICU. Although only two variables could be compared, they show benefits of this type of intervention in terms of reduction of hospital stay and in number of days to reach full oral feeding. However, the implementation methodology is very heterogeneous. This prevents the best method of stimulation intervention is known. Even so, those studies that used the PIOMI, refs. [37,38] as well as being the most current, demonstrate a significant improvement in motor function compared to the rest of the studies. This leads us to believe that PIOMI may be the best intervention to improve motor function in preterm infants.

Our results are consistent with previous studies [14,20,22,25,47]. We also observed that these benefits also occur with other types of therapy [20,28,48]. This implies that treatment through stimulation and touch is essential for babies admitted to the Neonatal Intensive Care Unit, as long as they do not expose babies to stress [10].

All studies that evaluated feeding parameters reported early acquisition of independent breastfeeding and improvements in sucking patterns, with the exception of sucking-swallowing coordination, where the results obtained were not considered significant [46]. These results should be complemented by other findings such as maturation in the developmental stages of sucking, as these are fundamental to understanding possibilities for improvement in the infant’s sucking skills [49]. In addition, it would be interesting to know how the combination of oral sensorimotor stimulation is complemented by other types of massage that improve gastric motility [13,48]. Of the studies that evaluated hospital stay, three [37,38,44] showed a significant decrease, while the other two [43,46] could not be considered relevant.

Variables classified as infant alertness, physiological data and breastfeeding skills did not achieve significant changes in the studies where they were evaluated [37,46].

As for the differences in the time and frequency of the sessions, there are no data that demonstrate greater benefits of one or the other. Even so, all the studies report positive results in the evaluation of the different variables. This indicates that this therapy will always be a reason for improvement in the development of premature infants.

The assessment of the long-term effects of the intervention plan was measured in only one study, [46] confirming that the subjects continue breastfeeding satisfactorily. It would be interesting for future studies to consider assessing this aspect. This would support that the benefits of the therapy do not only work in the short term, but are sustained over time.

Of the studies analysed and included in this review, none showed any type of contraindication or negative side effects during the intervention programme or afterwards once it has been completed. Furthermore, it is worth highlighting the low cost of this type of intervention programme, due to the fact that specific devices would not be needed for its implementation, but rather the figure of the physiotherapist. This would result in an improvement in the quality of care for this type of patient, without a high economic impact on the Neonatal Intensive Care Units, if the professional is already on staff.

Research to date on oral sensorimotor intervention as a strategy in the management of preterm infants is quite limited. In addition, there is a need for a greater variety of authors to conduct this type of study. The characteristics of the stimulation program were not specified in all studies. This makes it difficult to know which programmes have been applied and prevents replication of those with the best results.

No reviews have been found that specifically refer to the impact of oral sensorimotor stimulation on these babies. Our study appears to be the first to bring together the current literature on this type of approach. Therefore, it can be a tool for guidance and consultation on the different protocols of oral sensorimotor therapy in the treatment of preterm patients admitted to the NICU.

## 5. Conclusions

Oral sensorimotor stimulation is beneficial for the acquisition of independent oral feeding in preterm infants by decreasing the days of admission and the number of days to reach full oral feeding.

## Figures and Tables

**Table 1:** Search Strategies.

Search Strategy 1 TS = ((“Premature Birth” OR “Infant, Extremely Premature” OR “Infant, Premature”) AND “Mouth” AND (“Physical Therapy Modalities” OR “Physical Therapy Specialty” OR “Physical Therapy Department, Hospital” OR “Physical Therapist Assistants”))				
Database	Total Results	Filters and Inclusion Criteria	Elimination of Duplicates	Valid Results
PubMed	*n* = 4	*n* = 4	*n* = 4	*n* = 4
Scopus	*n* = 0	*n* = 0	*n* = 0	*n* = 0
WoS ^1^	*n* = 0	*n* = 0	*n* = 0	*n* = 0
Search Strategy 2 TS = ((“Premature Birth” OR “Infant, Extremely Premature” OR “Infant, Premature”) AND “Mouth”)				
Database	Total Results	Filters and Inclusion Criteria	Elimination of Duplicates	Valid Results
PubMed	*n* = 282	*n* = 23	*n* = 19	*n* = 3
Scopus	*n* = 427	*n* = 82	*n* = 73	*n* = 0
WoS	*n* = 527	*n* = 62	*n* = 53	*n* = 3
Search Strategy 3. Advanced Search. “Premature” for title and abstract, “Stretching, mobilisation, manipulation, massage” for Therapy, “Head or neck” for Body part y “Clinical trial” for Method.				
Database	Total Results	Filters and Inclusion Criteria	Elimination of Duplicates	Valid Results
PEDro ^2^	*n* = 2	*n* = 1	*n* = 1	*n* = 1
Search Strategy 4. Simple Search. “Oral sensorimotor intervention”				
Database	Total Results	Filters and Inclusion Criteria	Elimination of Duplicates	Valid Results
PEDro	*n* = 6	*n* = 0	*n* = 0	*n* = 0
Search Strategy 5. Simple Search. “Physiotherapy in premature infants”				
Database	Total Results	Filters and Inclusion Criteria	Elimination of Duplicates	Valid Results
PEDro	*n* = 12	*n* = 2	*n* = 2	*n* = 0
Search Strategy 6. Simple Search. “Oral sensorimotor intervention in preterm infants”				
Database	Total Results	Filters and Inclusion Criteria	Elimination of Duplicates	Valid Results
PEDro	*n* = 4	*n* = 4	*n* = 0	*n* = 0

^1^ WoS, Web of Science; ^2^ PEDro, Physiotherapy Evidence Database.

**Table 2:** Summary of the most relevant sample’s data.

Authors Country, Year	Sample (N) Groups (*n*)	Characteristics	Intervention
Fucile, S. Gisel, E.G. Lau, C. [41] USA, 2005	*n* = 32 EG: oral stimulation (*n* = 16). CG: control group (*n* = 16).	Mean age (wks)/Gender G1: 28.2, 7M:9F G2: 28.1, 6M:10F Inclusion: Gestational age (26–29 weeks), Tube feeding, No congenital malformations, No chronic diseases	Total duration: 10 days Assessment −1 to 2 oral/feedings/day −6 to 8 oral feedings/day G1: oral stimulation program, stroking the perioral and intraoral (15 min) G2: no intervention
Boiron, M. Da Nobrega, L. Roux, S. Henrot, A. Saliba, E. [39] France, 2007	*n* = 43 G1: Oral Stimulation + Oral support (*n* = 9). G2: Oral Stimulation (*n* = 11). G3: Oral support (*n* = 12). G4: Control group (*n* = 11).	Mean age (wks)/Gender G1: 31.3/M5:F4 G2: 31.1 M4:F7 G3: 31.6 M7:F5 G4: 31.1 M11:4F Inclusion: Gestational age (29–34 weeks), Naso-gastric tube feeding, No respiratory support in the preceding 48 h, Normal blood oxygen levels. Exclusion: Perinatal asphyxia, Intrauterine growth retardation, Severe jaundice with exsanguination transfusion, Cerebral lesions after severe grade III or IV intraventricular hemorrhage, Bronchopulmonary dysplasia were excluded	Total duration: 14 days. Assessment -D1: 1st day -D7: 4th day -D14: 14th day -D17: 17th day -D20: 20th day -D23: 23th day G1: Oral Stimulation+ oral support G2: Oral Stimulation G3: Oral Support G4: Control group Oral Stimulation: 12 min, once a day Oral Support during bottle feeding: 10 min, twice a day
Fucile, S. Gisel, E.G. [42] Canada, 2010	*n* = 75 G1: Oral stimulation (O) (*n* = 19). G2: Tactile/Kinesthetic stimulation (T/K) (*n* = 18) G3: Oral + Tactile/Kinesthetic stimulation (O+T/K) (*n* = 18) G4: Control group (*n* = 20)	Mean age (wks)/Gender G1: 29.6 ± 1.5, M12:F7 G2: 29.1 ± 2, M11:F7 G3: 29.0 ± 1.8, M3:F8 G4: 29.4 ± 1.9. M16:F4 Inclusion: Gestational age (26–32 weeks), Appropriate size for their GA, Tube feeding, No congenital malformations, No chronic diseases	Total duration: 10 days. G1: 15-min oral stimulation (O) intervention twice a day (total: 30 min per day) G2: T/K 15-min tactile/kinesthetic intervention (T/K) (stroking the body) twice a day (total: 30 min per day) G3: 15-min O +15-min T/K G4: Control group
Hwang, Y.-S. Vergara, E. Lin, C.-H. Coster, W.J. Bigsby, R. Tsai, W.-H. [36] Taiwan, 2010	*n* = 19 EG: oral stimulation CG: Control group	Mean age (wks)/Gender 28.1 ± 2.6,/7M:12F Inclusion: Gestational age (24–34), Inefficient feeders (<4 mL mL/milk in the first 5 min) Exclusion: Congenital anomalies affecting feeding and digestive function, Chromosomal or genetic problems, Medical instability	Total duration: 2 days EG: 5 min peri and intraoral stimulation CG: no intervention
Fucile, S. Gisel, E.G. McFarland, D.H. Lau, C. [40] Canada, 2011	*n* = 75 G1: Oral stimulation (O) (*n* = 19). G2:Tactile/Kinesthetic stimulation (T/K) (*n* = 18). G3: Oral + Tactile/Kinesthetic stimulation (O+T/K) (*n* = 18). G4: Control group (*n* = 20).	Mean age (wks)/Gender G1: 29.6 (0.4) .2, 12M:7F G2: 29.1 (0.3), 11M:7F G3: 29.0 (0.3), 10M:8F G4: 29.4 (0.1), 16M:4F Inclusion: Gestational age (26–32 weeks), Appropriate size for their GA, Tube feeding, No congenital malformations, No chronic diseases	Total duration: 10 days Assessment −1 to 2 oral/feedings/day(o/f/d)−3 to 5 o/f/d −6 to 8 o/f/d G1: 15-min oral stimulation (O) intervention twice a day (total: 30 min per day) G2: T/K 15-min tactile/kinesthetic intervention (T/K) (stroking the body) twice a day (total: 30 min per day) G3: 15-min O +15-min T/K G4: Control group
Fucile, S. McFarland, D.H. Gisel, E.G. Lau, C. [44] Canadá, 2012	*n* = 75 G1: Oral stimulation (O) (*n* = 19) G2:Tactile/Kinesthetic stimulation (T/K) (*n* = 18) G3: Oral + Tactile/Kinesthetic stimulation (O + T/K) (*n* = 18) G4: Control group (*n* = 20)	Mean age (wks)/Gender G1: 29.6 (0.4) .2, 12M:7F G2: 29.1 (0.3), 11M:7F G3: 29.0 (0.3), 10M:8F G4: 29.4 (0.1), 16M:4F Inclusion: Gestational age (26–32 weeks), Appropriate size for their GA, Tube feeding, No congenital malformations, No chronic diseases	Total duration: 10 days Assessment −1 to 2 oral/feedings/day(o/f/d)−3 to 5 o/f/d −6 to 8 o/f/d G1: 15-min oral stimulation (O) intervention twice a day (total: 30 min per day) G2: T/K 15-min tactile/kinesthetic intervention (T/K) (stroking the body) twice a day (total: 30 min per day) G3: 15-min O +15-min T/K G4: Control group
Bala, P. Kaur, R. Mukhopadhyay, K. Kaur, S. [38] India, 2016	*n* = 51 EG: Oro-motor stimulation (OMS) (*n* = 25) CG: Control group (*n* = 26).	Mean age (wks)/Gender EG: 30.9; 10M:15F CG: 30.3; 16M:10F Inclusion: Gestational age (28–34 weeks), Tube feeding Exclusion: Respiratory distress, Congenital malformations	Total duration from birth to discharge EG: OMS by mother five times a day before each feed CG: Kangaroo mother care and non-nutritive sucking
Fucile, S. Milutinov, M. Timmons, K. Dow, K. [45] Canada, 2018	*n* = 31 EG: oral sensorimotor stimulation(*n* = 16). CG: Control group (*n* = 15).	Mean age (wks)/Gender EG: 32.1 ± 1.6, 11M:5F CG: 31.2 ± 2.6, 13M:2F Inclusion: Gestacional age > 34 e., Appropriate size for GA, Tube feeding, Mother’s breastfeed intention Exclusion: Congenital malformations, Chronic medical complications, Bronchopulmonary, Dysplasia, Necrotizing enterocolitis, Intraventricular hemorrhage (IVH) grades III and IV, Periventricular leukomalacia, Congenital anomalies	Total duration: 10 days EG: 15-min oral sensorimotor stimulation once day. CG: 15-min sham intervention once day.
Arora, K. Goel, S. Manerkar, S Konde, N. Panchal, H. Hegde, D. Mondkar, J. [37] India, 2018	*n* = 30 EG: Premature Infant Oro-motor Intervention (PIOMI (*n* = 16) CG: Control group (*n* = 14).	Mean age (wks)/Gender EG: 30; 8M:8F CG: 30.5; 8M:6F Inclusion: Gestational age (28–34 weeks), Tube feeding Exclusion: Respiratory distress, Congenital malformations	Total duration: 7 days. Assessment: Baseline, 7 days EG: PIOMI three times a day CG: sham intervention
Ghomi, H. Yadegari, F. Soleimani, F. Knoll, B.L. Noroozi, M. Mazouri, A. [35] Iran, 2019	*n* = 30 EG: Premature Infant Oro-motor Intervention (PIOMI) (*n* = 15) CG: Control group (*n* = 15).	Mean age (days)/Gender EG: 197.50; 7M:8F CG: 197.60; 8M:7F Inclusion: Gestational age (26–29 weeks), Tube feeding, Apgar score of 6 at 5 min after birth Exclusion: Respiratory distress, Congenital malformations chromosomal abnormalities, Bronchopulmonary, Dysplasia, Necrotizing enterocolitis, Intraventricular hemorrhage (IVH) grades III and IV	Total duration: 10 days. Assessment: 1st, 4th, 8th and 10th days. EG: 5 min, PIOMI once a day CG: Control group
Aguilar-Rodriguez M. Leon-Castro JC. Alvarez-Cerezo M. Aledon-Andujar N. Escrig-Fernandez R. Rodríguez deDios-Benlloch J. Hervás-Marín D. Vento-Torre M. Spain, 2019	*n* = 46 EG: OSMS protocol (*n* = 24) CG: Control group (*n* = 22).	Mean age (wks)/Gender EG: 28.21 (1.33) 27M:19F CG: 197.60; 8M:7F Inclusion: Gestational age (25–30 weeks), Tube feeding Exclusion: Congenital anomalies affecting feeding, Pathologies affecting hemodynamic or cardiorespiratory stability, Suffering severe sepsis	Total duration:2 weeks, 20 sessions in total, except weekends EG: OSMS protocol, 10 min CG: Control group

**Table 3:** Summary of the most relevant outcomes and results.

Authors Country, Year	Outcomes	Results		
Fucile, S. Gisel, E.G. Lau, C. [41] USA, 2005	Nr days to reach full oral feeding	Mean (SD) **Nr days to reach full oral feeding** EG: 11 (4)/CG: 18 (7) *p* = 0.005		
	Overall intake (volume taken/volume prescribed %)	1 to 2 oral feedings/day **Overall intake:** EG: 80 (30)/CG: 50 (30) *p* = 0.001		6 to 8 oral feedings/day **Overall intake** EG: 89 (30)/CG: 67 (33) *p* = 0.06
	Rate of milk transfer (mL/min)	1 to 2 oral feedings/day **Rate of milk transfer** EG: 1.6 (0.7)/CG: 0.9 (0.6) *p* = 0.02		6 to 8 oral feedings/day **Rate of milk transfer** EG: 2.3 (1.0)/CG: 1.6 (0.9) *p* = 0.08
	Suction and expression amplitudes (Mikro-tip sensor)	1 to 2 oral feedings/day **Suction amplitude** EG: −31.9 (39.4) ^a^/CG: −27.6 (21.4) *p* = 0.47 **Expression amplitude** EG: 32.9 (36.6) CG: 7.9 (9.03) *p* = 0.0003		6 to 8 oral feedings/day **Suction amplitude** EG: −38.9 (25.2)/CG: −38.5 (26.5) *p* = 0.36 **Expression amplitude** EG: 12.4 (8.8)/CG: 9.7 (9.4) *p* = 0.01
Boiron, M. Da Nobrega, L. Roux, S. Henrot, A. Saliba, E. [39] France, 2007	Nr days to reach full oral feeding	ANCOVA (means (SEM)) **Nr days to reach full oral feeding** F [3,39] = 3.71, *p* < 0.00 (group)		
	Sucking pressure	Effects G2, (D1, D7, D14) **Sucking pressure** F [3,38] = 20.30, *p* < 0.001 (group) group×time interaction F [6,76] = 10.96, *p* < 0.0001, ε = 0.81		Effects G1, (D17, D20, D23) **Sucking pressure** F [3,38] = 18.49, *p* < 0.001 (group) group × time interaction F [3,114] = 4.19, *p* = 0.014, ε = 0.77
	Succking activity	Effects G2, (D1, D7, D14) **Suking activity** F [3,38] = 11.77, *p* < 0.001 (group) group × time interaction F [6,76] = 8.35, *p* < 0.0001, ε = 0.92		Effects G1, (D17, D20, D23) **Suking activity** F [3,38] = 11.03, *p* < 0.001 (group) group × time interaction F [3,114] = 9.58, *p* < 0.001, ε = 0.87
	Daily bottle feeds	**Daily bottle feeds** F [3,38] = 24.23, *p* < 0.001 (group) group × time interaction F [2,76) = 9.13, *p* < 0.0005, ε = 0.88		
	Daily milk ingestion	**Daily milk ingestion** F [3,38] = 8.72, *p* = 0.002 (group) group×time interaction F [6,76] = 3.13, *p* = 0.01, ε = 0.94		
		No significant difference between the four groups for birth-weight or GA at birth at the outset and end of the protocol		
Fucile, S. Gisel, E.G. [42] Canada, 2010	Daily weight gain	One-way ANOVA **Daily weight gain** During intervention G1: 17.4 ± 6.1 G2: 17.6 ± 3.8 G3: 15.7 ± 4.6 G4: 12.6 ± 5.9 *p* = 0.014		**Daily weight gain** After intervention G1: 16.1 ± 6.9 G2: 15.1 ± 7.4 G3: 13.1 ± 7.3 G4: 14.3 ± 5.4 *p* = 0.583
	Test of Infant Motor Performance (TIMP).	**TIMP** G1: 34.6 ± 4.5 G2: 36.7 ± 4.0 G3: 36.9 ± 4.7 G4: 30.2 ± 8.2 *p* = 0.003		
Hwang, Y.-S. Vergara, E. Lin, C.-H. Coster, W.J. Bigsby, R. Tsai, W.-H. [36] Taiwan, 2010	Intake rate	**Intake rate, initial 5 min:** EG: 2.3 (1.6) CG: 1.7 (1.1), *p* = 0.0021		
	Feeding screening assessment Feeding duration (min)	Feeding parameters: no differences except to: **Feeding start. Alertness categories** Day 1. Extreme to middle EG vs. CG, *p* = 0.0016 Day 2. Extreme to middle EG vs. CG, *p* = 0.0016		
	Sucking frecuency (sukcs/min) Mean ingested per suck (mL/suck) % volume ingested (mL/min) Neonatal Behavioral Assessment Scale’ (NBAS). Oxygen saturation: MARS pulse oximeter	No more significant differences were found.		
Fucile, S. Gisel, E.G McFarland, D.H. Lau, C. [40] Canada, 2011	No. days to reach full oral feeding Length of hospital stay (d)	**No. days to reach full oral feeding** G1: 11.1 (0.8) G2: 11.4 (0.8) G3: 10.0 (0.8) G4: 20.7 (1.5) *p* ≤ 0.001		
	Volume transfer (mL/min)	**Volume transfer** F [3,69] = 17.018; *p* < 0.00 (group) Group × time interaction F [6,141] = 4.879; *p* < 0.001		
	Rate of transfer (mL/min)	**Rate of transfer** F [3,69] = 4.634; *p* = 0.009		
	Volume loss (mL/min)	**Volume loss** F [3,69] = 4.567; *p* = 0.00 (group)		
Fucile, S. McFarland, D.H. Gisel, E.G. Lau, C. [44] Canadá, 2012	Sucking stage scale (Lau et al.)	One-way ANOVA 1 to 2 oral feedings/day **Stage of sucking** G1: 3.3 (0.1) vs. control group *p* = 0.003 G2: 3.1 (0.1) G3: 3.2 (0.1) G4: 2.8 (0.1) F [3,69] = 5.222, *p* = 0.00 (group) group × time interaction F [6,141] = 2.930, *p* = 0.02	3 to 5 oral feedings/day **Stage of sucking** G1: 3.3 (0.1) G2: 3.2 (0.1) G3: 3.1 (0.1) G4: 3.0 (0.1)	6 to 8 oral feedings/day **Stage of sucking** G1: 3.7 (0.1) vs. G2, G3 and G4: *p* < 0.039 vs. control group *p* = 0.003 G2: 3.3 (0.1) G3: 3.2 (0.1) G4: 3.1 (0.1) F [3,69] = 5.222, *p* = 0.00 (group) group × time interaction F [6,141] = 2.930, *p* = 0.02 There were no significant in suck–swallow coordination
	Suck to swallow ratio	1 to 2 oral feedings/day **Suction amplitude** G1: −35.3 (0.5) G2: −34.8 (6.3) G3: −28.8 (4.5) G4:−24.0 (3.4) F3 = 694.804, *p* = 0.011 (group)	3 to 5 oral feedings/day **Suction amplitude** G1: −53.4 (5.5) G2: −42.4 (8.7) G3: −41.8 (8.1) G4: −35.9 (5.0) F [3,69] = 4.804, *p* = 0.011 (group)	6 to 8 oral feedings/day **Suction amplitude** G1: −46.4 (5.5) G2: −46.4 (6.7) G3: −46.7 (8.8) G4: −38.3 (5.8) F [3,69] = 4.804, *p* = 0.011 (group)
	Swallow–respiration	1 to 2 oral feedings/day **Expression amplitude** G1: 6.5 (1.9) G2: 1.9 (0.6) G3: 6.7 (1.5) G4: 1.5 (0.3) F [3,69] = 8.347, *p* = 0.006 (group) Group × time F [2,138] = 8.434, *p* = 0.007	3 to 5 oral feedings/day **Expression amplitude** G1: 3.4 (1.2) G2: 1.9 (0.4) vs. G4 *p* = 0.034 G3: 3.4 (1.0) G4: 3.7 (0.6)	6 to 8 oral feedings/day **Expression amplitude** G1: 15.7 (2.6) vs. G2, G3 and G4, *p* < 0.003 G2: 3.2 (0.7) G3: 2.9 (0.9) G4: 2.5 (0.5) F [3,69] = 8.347, *p* = 0.006 (group) Group × time F [2,138] = 8.434, *p* = 0.007 There were no significant in suck–swallow coordination.
Bala, P. Kaur, R. Mukhopadhyay, K. Kaur, S. [38] India, 2016	No. days to reach partial and full spoon feed Total volume of milk by spoon at each feed Time required to complete full spoon feed Partial direct breast feed at discharge	**Transition time** (Median (IQR) -Partial spoon feed EG: 5 (3–9.5) CG: 10 (5–15) *p* = 0.006 -Full spoon feed EG: 7 (5–14.5) CG: 12.5 (7–21) *p* = 0.03)		**Feed mood at discharge** -Partial breast/spoon EG: 14 (56) CG: 8 (31) *p* = 0.01 -Only spoon feed EG: 10 (40) CG: 18 (69) *p* = 0.03
Fucile, S. Milutinov, M. Timmons, K. Dow, K. [45] Canada, 2018	No. days to reach full oral feeding	**No. days to reach full oral feeding** EG:10.7–2.1 CG:19.3–3.6 *p* = 0.01		
	Acquisition of breastfeeding	**Acquisition of breastfeeding** EG:11 CG: 5 *p* = 0.049		
	Duration of hospital stay	**Duration of hospital stay** EG: 33.0 (15.7) CG: 43.2 (27.39) *p* = 0.054		
	PIBBS score	There were no significant in PIBBS and breastfeeding duration follow-up.		
Arora, K. Goel, S. Manerkar, S Konde, N. Panchal, H. Hegde, D. Mondkar, J. [37] India, 2018	Duration of hospital stay	**No. days to reach full oral feeding** EG: 4.0 (0.8) CG: 6.6 (1.0) *p* < 0.001		
	Neonatal Oro-Motor Assessment Scale (NOMAS)	**Improvement in NOMAS score** EG: 9.3 (1.7) CG: 4.8 (1.5) *p* < 0.001		
	Time to reach full independent wati spoon feeds Weight gain after intervention	No significant difference in terms of hospital stay after enrolment and weight gain at discharge.		
Ghomi, H. Yadegari, F. Soleimani, F. Knoll, B.L. Noroozi, M. Mazouri, A. [35] Iran, 2019	No. days to reach full oral feeding	**No. days to reach full oral feeding** EG: 8.07 (2.58) CG: 14.33 (4.70) *p* < 0.001		
	Duration of hospital stay	**Duration of hospital stay** EG: 37.13 (11.70) CG: 46.60 (11.35) *p* = 0.03		
	Weight gain after intervention	No significant difference in terms of weight gain at discharge.		
Aguilar-Rodriguez M. Leon-Castro J.C. Alvarez-Cerezo M. Aledon-Andujar N. Escrig-Fernandez R. Rodríguez deDios-Benlloch J. Hervás-Marín D. Vento-Torre M. Spain, 2019	No. days to reach full oral feeding	Mean (SD) Median (1st, 3r) **No. days to reach full oral feeding** EG:36 (26.75, 40) CG: 42.5 (34.25, 47.75) *p* = 0.0013		
	No. days 100% intake	**No. days 100% intake** EG: 26.33 (10.11) CG: 32.64 (9.44) *p* = 0.040		
	Duration of hospital stay	**Duration of hospital stay** EG: 43 (39, 50.25) CG: 50 (43.25, 59.75) *p* = 0.028		
	Weight gain after intervention	No significant difference in terms of weight gain at discharge.		

In the results column, the outcome variable has been highlighted in bold and the times at which the variable was measured have been underlined.

**Table 4:** PEDro scale of methodological quality.

Study, Year	PEDro Scale Items											Total
	Item 1	Item 2	Item 3	Item 4	Item 5	Item 6	Item 7	Item 8	Item 9	Item 10	Item 11	
Fucile, 2010 [43]	1	1	0	1	0	0	1	0	0	1	1	5
Fucile, 2018 [45]	1	1	0	0	0	0	1	0	0	1	1	4
Fucile, 2012 [44]	1	1	0	0	0	0	0	1	0	1	1	4
Fucile, 2011 [40]	1	1	0	1	1	1	0	1	0	1	1	7
Ghomi, 2019 [35]	1	1	0	1	1	0	1	1	0	1	1	7
Borion, 2007 [39]	0	1	0	1	0	0	0	0	0	1	1	4
Hwang, 2010 [36]	1	1	0	1	1	0	0	1	0	1	0	5
Arora, 2018 [37]	1	1	1	1	1	0	1	1	0	1	1	8
Bala, 2016 [38]	1	1	1	1	1	0	0	1	0	1	0	6
Fucile, 2005 [41]	1	1	0	0	0	0	0	1	0	1	1	4
Aguilar, 2020 [44]	1	1	0	0	0	0	1	1	0	1	1	6

**Table 5:** Publication bias. Begg and Egger test.

Variable	Begg (*p*)	Egger (*p*)
Duration of hospital stay	*p* = 0.0085	*p* = 0.0003
Number of days to reach full oral feeding	*p* = 0.7341	*p* = 0.2092
